# Psychological Impact of Vehicle Exhaust Exposure: Insights from an Animal Model

**DOI:** 10.1038/s41598-017-08859-1

**Published:** 2017-08-16

**Authors:** Ankita Salvi, Gaurav Patki, Hesong Liu, Samina Salim

**Affiliations:** Department of Pharmacological and Pharmaceutical Sciences, College of Pharmacy, University of Houston, Houston, TX-77204 USA

## Abstract

Air pollution resulting from exhaust emissions of vehicles has risen in the recent years, reportedly causing major adverse effects on the heart, lungs and the brain. Though respiratory and cardiovascular effects of these emissions are well identified, psychological and neurobiological complications of prolonged exposure to vehicle emissions remain unknown. Pro-oxidants are considered as major constituents of vehicle emissions. This is important considering causal link between oxidative stress and behavioral and cognitive impairments. We hypothesized that prolonged exposure to pro-oxidants in vehicle emissions result in behavioral and cognitive deficits. We developed a simulated vehicle exhaust exposure model in rats. The model used a simulated mixture of vehicle exhaust that comprised of pro-oxidant constituents of exhaust, namely, carbon dioxide (13%), carbon monoxide (0.68%) and nitrogen dioxide (1000 ppm) in air. Rats were exposed either to a high (1:10 dilution) or low (~1:1000 dilution) physiologically relevant dose of simulated mixture in air for two weeks in separate experiments followed by a comprehensive behavioral and cognitive analysis. We observed that prolonged exposure to pro-oxidants in vehicle exhaust increased anxiety-and depression-like behavior as well as led to impaired memory in rats. This is important preclinical evidence, particularly relevant to human population exposed to high vehicular traffic.

## Introduction

Air pollution is one of the serious environmental hazards that has resulted from increased urbanization. According to a report by the World Health Organization, air pollution due to gasoline and diesel emissions from internal combustion engines of automobiles, trucks, locomotives and ships leads to 800,000 premature deaths annually from pulmonary, cardiovascular and neurological complications. Effect of vehicle exhaust exposure on heart and lungs is well identified^1, 2^. However adverse effects of these emissions on the brain and its psychological impact has been ignored. Recent surveys suggest that individuals living and working in areas of heavy vehicular traffic generally suffer from mental comorbidities such as anxiety, depression and learning-memory impairment, thus making air pollution due to vehicle exhaust, a significant contributor to behavioral and cognitive deficits^3^. Here, we examined vehicle exhaust-induced behavioral and cognitive alterations in rats using a simulated vehicle exhaust exposure (SVEE) model. This is the first systematic study to utilize a unique and a highly innovative animal model, and comprehensively test the impact of vehicle exhaust exposure on behavioral and learning-memory functions in rats.

The composition of vehicle exhaust primarily comprises of gases such as carbon dioxide (CO_2_), carbon monoxide (CO) and nitrogen dioxide (NO_2_) as well as hydrocarbons and particulate matter^4, 5^. Out of these, CO_2_, CO and NO_2_ are pro-oxidant in nature^6^. Thus prolonged exposure to these gases could lead to increase in levels of oxidative stress in the body. Our previous studies have established a causal link between increase in oxidative stress in the brain and behavioral as well as cognitive deficits^7–10^. Therefore, it is possible that prolonged exposures to pro-oxidants from vehicle exhaust cause elevation of oxidative stress levels in the brain, eventually resulting in behavioral and learning-memory impairments.

**Figure 1:**
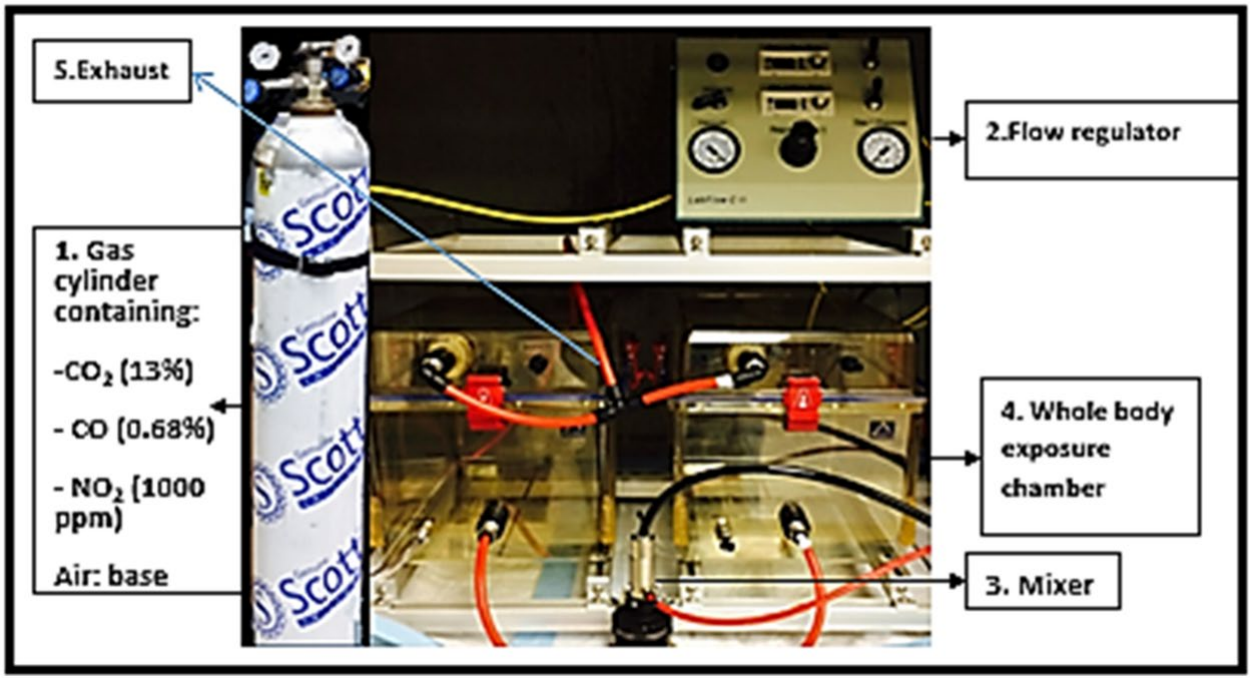
Simulated vehicle exhaust exposure (SVEE) model apparatus. SVEE consists of a whole body exposure system with two chambers fitted with tubing systems to allow inflow and outflow of gaseous mixtures. There is a cylinder that contains simulated mixture of vehicle exhaust. Gaseous mixture from the cylinder is blended with air in the mixer and then flow of the mixture is controlled using a flow regulator. Rats were subjected to SVEE in this apparatus.

In this study, we developed a novel SVEE model (Fig. 1). This is the first animal model of its kind and of tremendous value for behaviorists, toxicologists and psychologists. Basically, Sprague Dawley (SD) rats were subjected to: high dose brief exposure (1:10 dilution of simulated vehicle exhaust to air) and low dose prolonged exposure (~1:1000 dilution of simulated vehicle exhaust to air) for a duration of two weeks. This level of vehicle exhaust corresponds to the daily level of vehicle exhaust exposure experienced by people living in close proximity to highways for roughly 1.5 years^11, 12^. Following exposure, behavioral and learning-memory function analysis was performed to determine the psychological impact of vehicle exhaust on rats.

## Results

### High dose brief and low dose prolonged SVEE did not alter body weight, food and water intake in exposed rats

**Figure 2:**
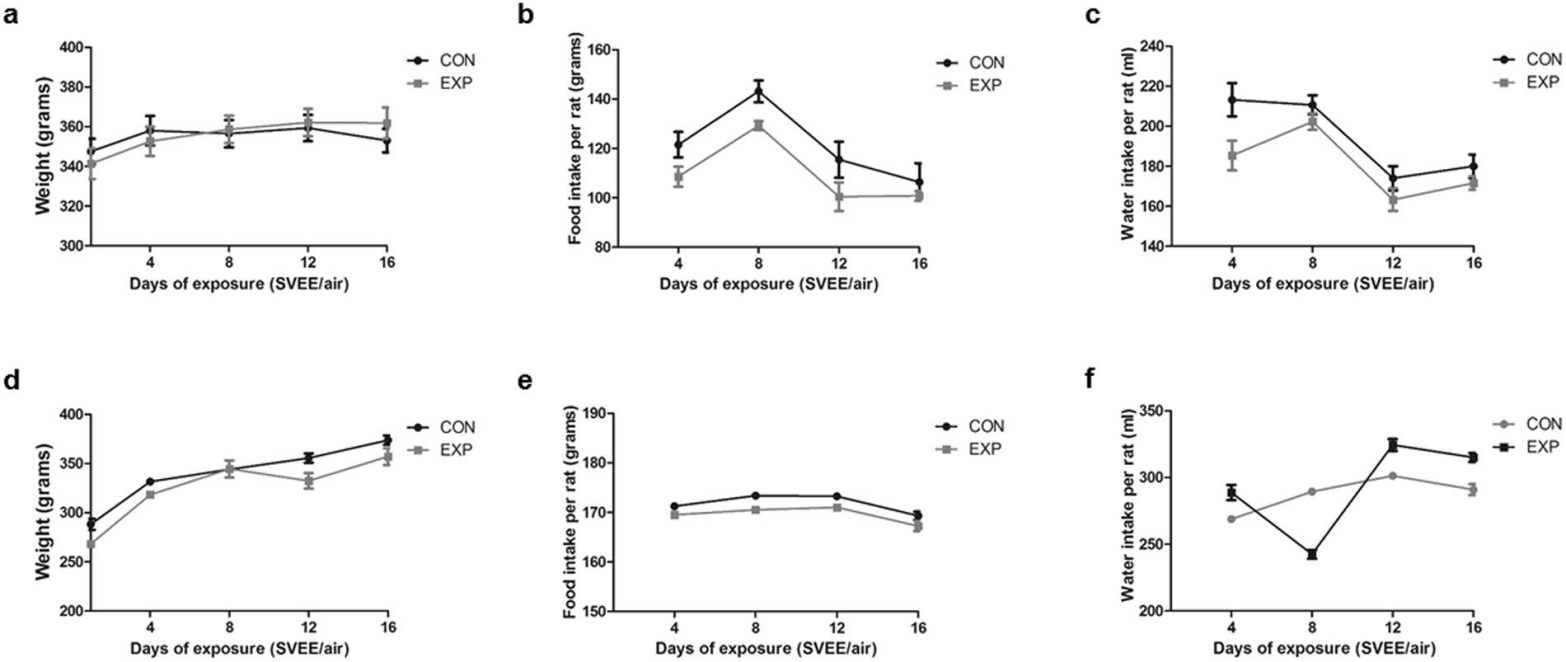
General body parameters following SVEE. No significant difference in weight gain, high dose brief (**a**), low dose prolonged (**d**) SVEE; food intake, high dose brief (**b**), low dose prolonged (**e**) SVEE; and water intake, high dose brief (**c**), low dose prolonged (**f**) SVEE following 2 weeks. Values are means ± SEM, n = 8 rats/group.

Body weight [high dose: F(4, 60) = 4.039, p = 0.1025; low dose: F(4, 60) = 6.557, p = 0.0626], food [high dose: F(3, 45) = 8.673, p = 0.0546; low dose: F(3, 45) = 8.208, p = 0.0587] and water intake [high dose: F(3, 45) = 6.540, p = 0.0787; low dose: F(3, 45) = 1.546, p = 0.3646] between control, exposed to normal air (CON) and exposed, exposed to simulated vehicle exhaust (EXP) rats remained unchanged following high dose brief and low dose prolonged SVEE (Fig. 2a–f). This suggests that exposure to vehicle exhaust did not alter general body parameters.

### High dose brief and low dose prolonged SVEE led to increased anxiety-like behavior in exposed rats

**Figure 3:**
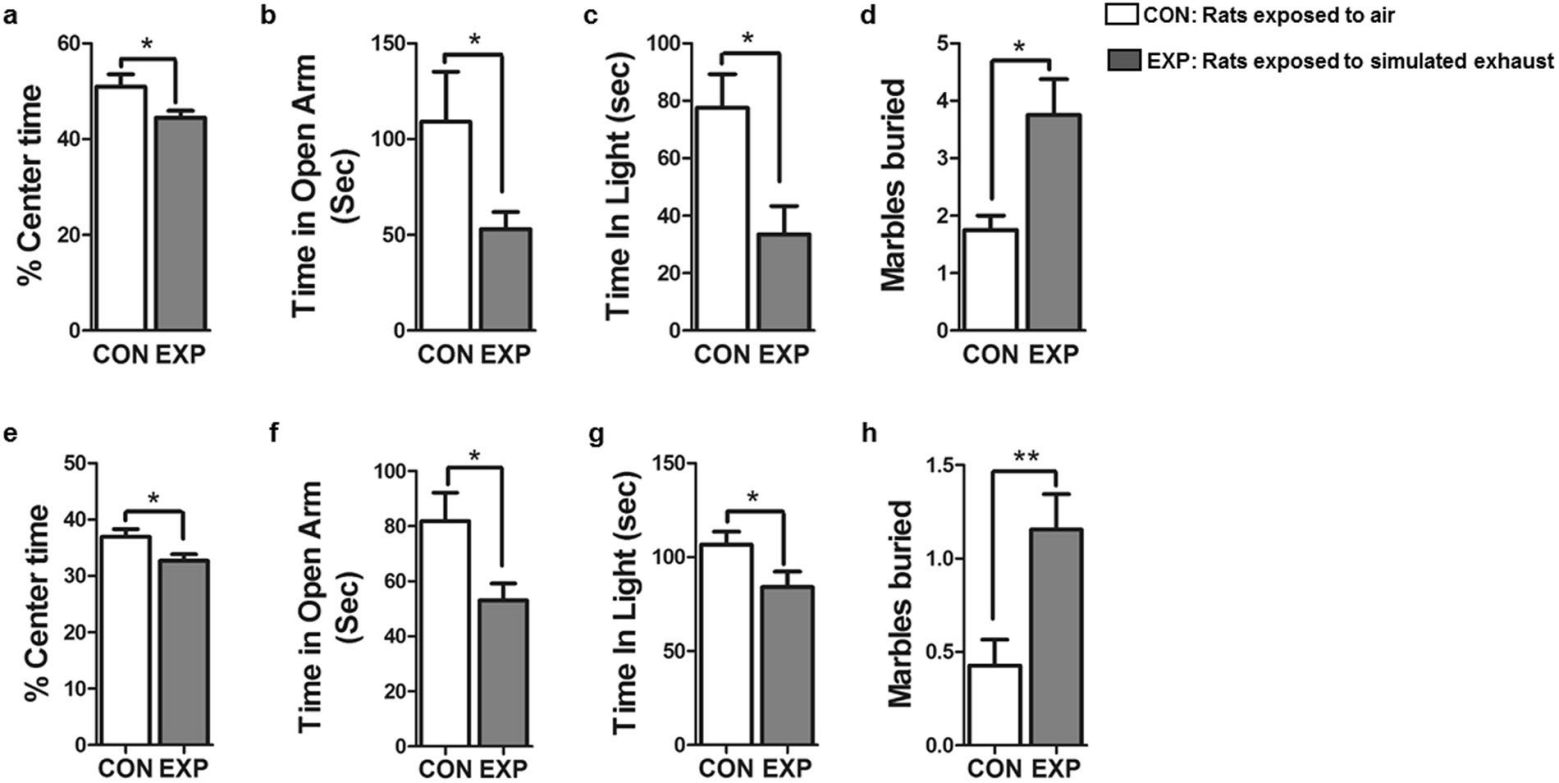
Examination of anxiety-like behavior (**a**–**h**) in rats subjected to high dose brief ((**a**–**d**), n = 9 rats/group) and low dose prolonged (**e**–**h**, n = 13 rats/group) 2 week SVEE. Anxiety-like behavior was assessed using open-field (**a**,**e**), elevated-plus maze (**b**,**f**), light-dark (**c**,**g**), and marble burying (**d**,**h**) tests. (*) Significantly different from control, p < 0.05, (**) significantly different from control p < 0.01. Bars represent means ± SEM.

In the Open Field Test (OFT), EXP rats spent significantly less time in the center as compared to CON rats following high dose brief SVEE (Fig. 3a, CON: 50.92 ± 2.60, EXP: 44.47 ± 1.43, −12%, p = 0.0429, t = 2.243, df = 16). Similar observation was made in EXP rats following low dose prolonged SVEE (Fig. 3e, CON: 36.94 ± 1.35, EXP: 32.68 ± 1.19, −11%, p = 0.0270, t = 2.356, df = 24).

In the Elevated Plus Maze (EPM) test, EXP rats spent significantly less time in the open arms of the EPM apparatus as compared to CON rats, following both high dose brief (Fig. 3b, CON: 109.0 ± 26.22, EXP: 53.0 ± 8.91, −51%. p = 0.0433, t = 2.313, df = 16) and low dose prolonged (Fig. 3f, CON: 81.91 ± 10.29, EXP: 53.00 ± 6.179, −34%, p = 0.0299, t = 2.348, df = 24) SVEE.

In the Light Dark (LD) test, EXP rats spent significantly less time in the lit compartment of the LD box as compared to CON rats following both high dose brief (Fig. 3c, CON: 77.57 ± 11.75, EXP: 33.56 ± 9.77, −56%, p = 0.0116, t = 2.901, df = 16) and low dose prolonged (Fig. 3g, CON: 106.7 ± 6.85, EXP: 84.0 ± 8.29, −21%, p = 0.0475, t = 2.112, df = 24) SVEE.

Similarly, in the Marble Burying (MB) test, EXP rats buried significantly more number of marbles as compared to CON rats following high dose brief (Fig. 3d, CON: 1.75 ± 0.25, EXP: 3.75 ± 0.62, +114%, p = 0.0255, t = 2.954, df = 16) and low dose prolonged (Fig. 3h, CON: 0.42 ± 0.13, EXP: 1.15 ± 0.19, +173%, p = 0.0045, t = 3.118, df = 24) SVEE.

In summary, both high dose brief and low dose prolonged 2 week SVEE led to increased anxiety-like behavior in EXP rats as examined using four different parameters of anxiety-like behavior.

### High dose brief and low dose prolonged SVEE led to increased depression-like behavior in exposed rats

**Figure 4:**
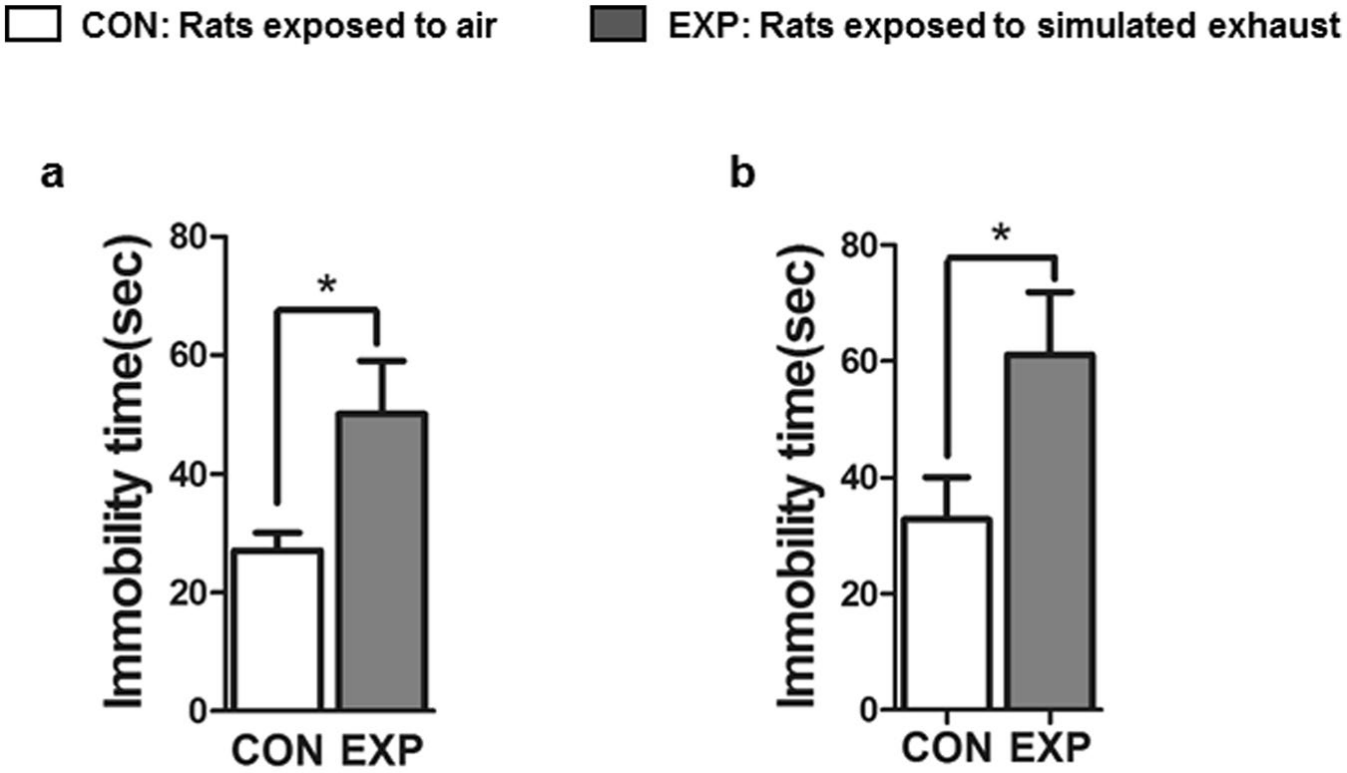
Examination of depression-like behavior (**a**,**b**) in rats subjected to high dose brief (**a**, n = 9 rats/group) and low dose prolonged (**b**, n = 13 rats/group) 2 week SVEE. Depression-like behavior was examined using forced swim test. (*) Significantly different from control p < 0.05. Bars represent means ± SEM.

In the Forced Swim Test (FST), EXP rats spent significantly more time immobile in the water tank as compared to CON rats, following both high dose brief (Fig. 4a, CON: 27.11 ± 3.02, EXP: 50.17 ± 8.86, +85%, p = 0.0131, t = 2.872, df = 16) and low dose prolonged (Fig. 4b, CON: 32.85 ± 7.24, EXP: 61.08 ± 10.79, +90%, p = 0.0378, t = 2.204, df = 24) SVEE. This suggests increased depression-like behavior following SVEE.

### High dose brief and low dose prolonged SVEE led to impaired memory in exposed rats

**Figure 5:**
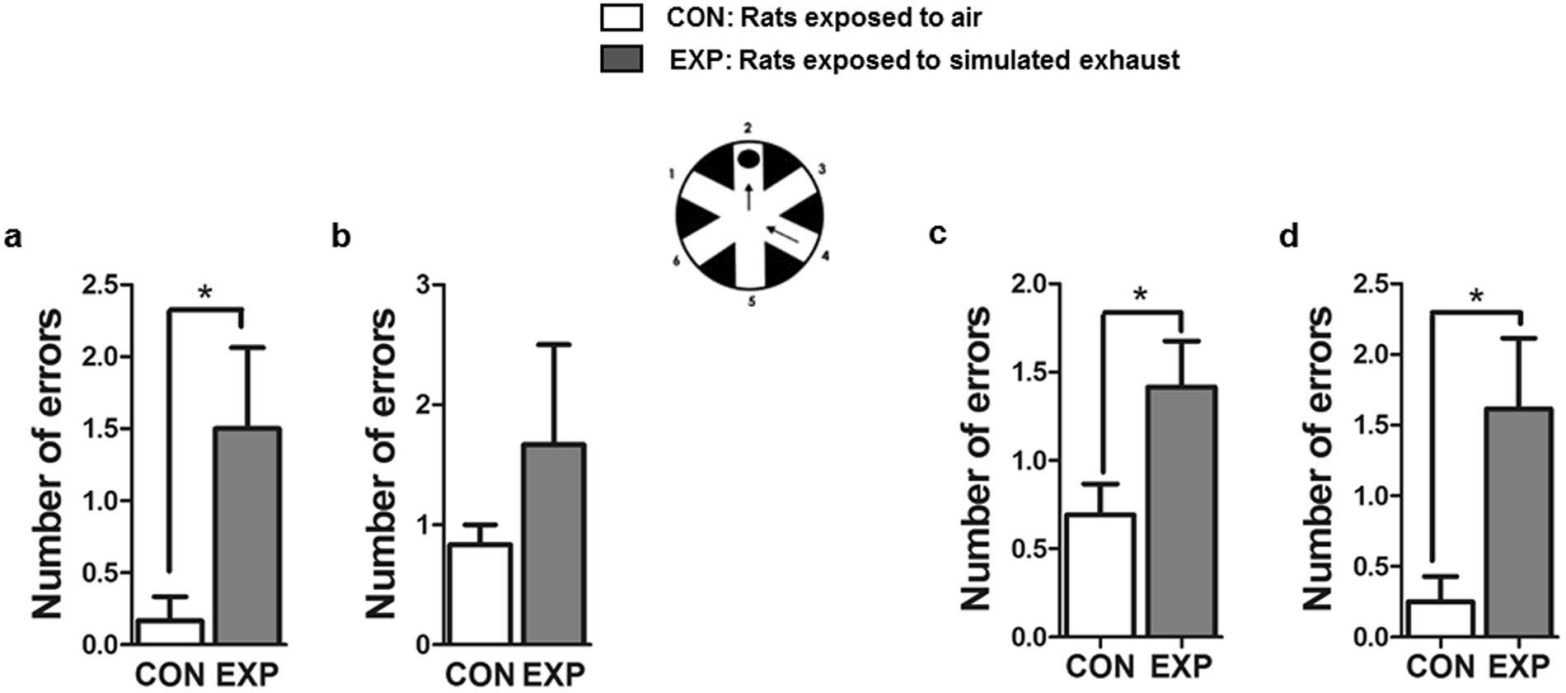
Examination of learning-memory function in rats subjected to high dose brief (**a**,**b**; n = 9 rats/group) and low dose prolonged (**c**,**d**; n = 13 rats/group) 2 week SVEE. Short-term (**a**,**c**) and long-term (**b**,**d**) memory was assessed by using 12 radial arm water maze (RAWM) trials. The RAWM apparatus is shown as inserts containing a circular water pool with 6 swim paths. (*) Significantly different from control p < 0.05. Bars represent means ± SEM.

In the Radial Arm Water Maze (RAWM) test, EXP rats made more number of errors in locating the hidden platform as compared to CON rats in short term memory (STM) test (Fig. 5a, CON: 0.16 ± 0.16, EXP: 1.5 ± 0.56, +837%, p = 0.0464, t = 2.272, df = 16) following high dose brief SVEE. There was not a significant difference in the number of errors between CON and EXP rats in long term memory (LTM) test (Fig. 5b, CON: 0.83 ± 0.16, EXP 1.66 ± 0.83, p = 0.4383, t = 0.799, df = 16). Following low dose prolonged SVEE, EXP rats made more errors in locating the hidden platform as compared to CON rats in both STM test (Fig. 5c, CON: 0.69 ± 0.17, EXP: 1.41 ± 0.25, +104%, p = 0.0280, t = 2.346, df = 24) and LTM test (Fig. 5d, CON: 0.25 ± 0.17, EXP: 1.61 ± 0.5, +544%, p = 0.0207, t = 2.485, df = 24).

Therefore, high dose brief SVEE impaired only short term memory in exposed rats, however low dose prolonged SVEE led to both short term and long term memory impairment.

## Discussion

In this study, we have examined neurobehavioral consequences of prolonged exposures to pro-oxidants in vehicle exhaust using a rat model of simulated vehicle exhaust exposure. *Firstly*, the simulated model was developed and optimized to mimic actual vehicle exhaust exposure condition in laboratory setting. Vehicle exhaust primarily comprises of nitrogen (71.5%), oxygen (0.7%), water (13.1%), CO_2_ (13.5%) and 1.1% of hazardous pollutants such as NO_2_, CO, hydrocarbons and particulate matter^4, 5^. Out of these, CO_2_, NO_2_ and CO are pro-oxidant in nature^6^. Since our aim was to investigate the effect of exposure to pro-oxidants on behavior and cognition, we used a simulated mixture of pro-oxidant gases from vehicle exhaust in our studies. Hydrocarbons and particulate matter present in vehicle exhaust are not pro-oxidants and are associated with irreversible adverse health effects such as cancer, cardiovascular and respiratory complications^13, 14^. Therefore, a major technical consideration in not including hydrocarbons and particulate matter in our exposure composition was to avoid interference of pulmonary (asthma-like conditions) and pro-cancerous effects with behavior. For example, it would become impossible for us to delineate whether a rat fails a learning-memory task or avoids movement due to high anxiety, poor cognition or simply due to increased hydrocarbon/particulate matter-induced breathlessness or cancer-related sickness. Hence, even though hydrocarbons and particulate matter are hazardous constituents of vehicle exhaust, our simulated exhaust comprised of the pro-oxidant gases, namely, CO_2_, CO and NO_2_ at concentrations as present in raw gasoline exhaust. The mixture so prepared was then provided to rats in whole body exposure chambers wherein the flow of gases was regulated at an optimum speed needed for ventilation of the chambers using flow modulators.

We performed our studies at two doses of the simulated exhaust since we wanted to compare the variation in response based on high dose brief exposure versus a low dose prolonged exposure. Therefore, 1:10 (high dose) and ~1:1000 (low dose) dilutions of simulated vehicle exhaust with air were used. The high dose exposure was provided for 30 min daily (brief) whereas the low dose exposure was provided for 5 h daily (prolonged). These levels of vehicle exhaust correspond to the daily level of vehicle exhaust exposure experienced by people living in close proximity to highways^11^. Both types of studies were performed for 2 continuous weeks, which is a sub-chronic duration of exposure in rats. Comparable behavioral and cognitive response was observed in rats at both doses. Levels of dilutions and duration of exposures were titrated in our lab and was also guided by another published report^15^.

*Secondly*, no change in body growth parameters such as weight gain, food intake and water intake was observed during the two weeks of exposure. Thus, exposure to pro-oxidants in vehicle exhaust does not alter daily body parameters or cause any sickness behavior. *Thirdly*, a significant increase in anxiety-like behavior was observed in rats exposed to vehicle exhaust. This was indicated by tests performed to measure anxiety-like behavior, namely, OFT, LD, EPM and MB tests. This behavior was consistent in both studies suggesting that exposure to pro-oxidants elevates levels of anxiety in rats at both high dose brief and low dose prolonged exposures. This effect was also extended to depression-like behavior which was observed to be elevated in rats exposed to simulated vehicle exhaust at both doses as indicated by FST. One point to be considered here is that we used a modified version of FST that lacked a pre-test phase where rats are dropped in the water-filled cylindrical tank of FST for 10 min before the actual test phase. This was done to avoid interference of pre-test phase associated stress on other behavior tests and to exclusively study effect of SVEE-associated stress on behavior and learning-memory function. Although, various groups including our own have used this version of FST to assess depression-like behavior and observed the test to be highly sensitive^16–18^, it is considered to have limited validation as a sole test to measure depression-like behavior by some groups and hence should be treated with caution.

*Finally*, exposure to simulated vehicle exhaust also impaired learning-memory function in rats. Rats exposed to exhaust made more errors in locating hidden platform in RAWM test which indicates that their working memory was impaired. Thus, exposure to pro-oxidants altered working memory in the rats. Again, these alterations in cognition were observed at both dose levels. These observations have high clinical relevance as these data provide causal links between vehicle emissions and neurobehavioral and cognitive deficits. This is critical information considering limited knowledge regarding the negative impact and adverse effects of vehicle emissions on the brain and its psychological consequences. This also warrants attention to individuals living and working in areas of heavy vehicular traffic who might be susceptible to developing anxiety, depression and learning-memory impairment.

**Figure 6:**
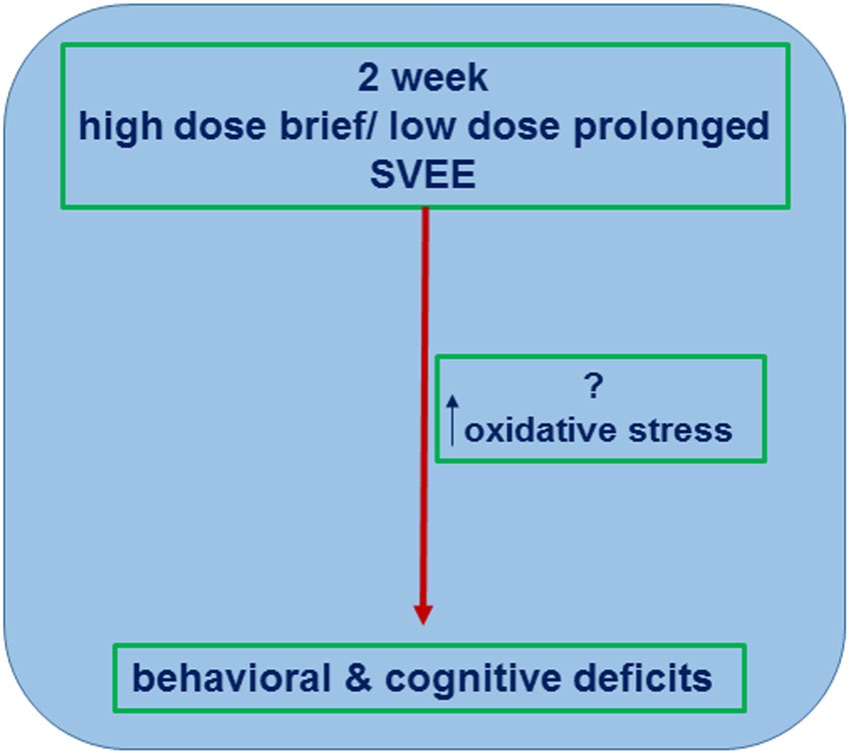
Schematic representation of the events potentially responsible for SVEE-induced behavioral and cognitive deficits.

The mechanistic basis for these behavioral and cognitive alterations are not clearly understood, however molecular changes in brain regions of pre-frontal cortex, hippocampus and amygdala seem plausible. Previous studies from our lab have suggested a causal link between increase in levels of oxidative stress and the consequent buildup of free radical species in pre-frontal cortex, hippocampus and amygdala and behavioral and cognitive deficits^7, 9^. Pre-frontal cortex and hippocampus are known to regulate working memory and cognition^19, 20^, whereas amygdala is reported to play a significant role in regulating emotions^21, 22^. Thus it is possible that elevated levels of free radicals in these regions trigger downstream molecular pathways that subsequently result in behavioral and cognitive alterations (Fig. 6). Future studies will confirm the role of oxidative stress in SVEE-induced behavioral and cognitive alterations.

In conclusion, our study proposes a novel and translationally relevant model to investigate the effect of prolonged exposures to pro-oxidants in vehicle exhaust on rats in a laboratory setting. We have established that prolonged SVEE results in increased anxiety-like, depression-like behavior and impaired memory function. This is the first pre-clinical study to report behavioral and cognitive impairment in response to a physiologically relevant dose of vehicle pro-oxidant exposure. This innovative model can not only be used to address behavioral and psychological effects of a variety of laboratory simulations but it can also be used to probe underlying neurobiological mechanisms. Our hypothesis is that SVEE-induced behavioral and cognitive alterations are a consequence of SVEE-induced increase in oxidative stress in the brain.

## Methods

### Experimental Design

Adult male SD rats (225-250 g, 10-12 weeks old) were divided into 2 groups: control (CON): exposed to normal air; and exposed (EXP): exposed to simulated vehicle exhaust. There were two independent studies performed separately to measure the effect of two different doses of SVEE:

- **High dose brief exposure:** 1:10 dilution of exhaust exposure for 2 weeks, daily 30 min
- **Low dose prolonged exposure:** ~1:1000 dilution of exhaust exposure for 2 weeks, daily 5 h

The purpose of using two dose levels of vehicle exhaust was to compare if the SVEE-induced behavioral and cognitive alterations vary depending on whether the daily exposure occurs at a high dose for shorter duration (1:10, 30 min) or low dose but longer duration (~1:1000, 5 h). Both high dose brief exposure and low dose prolonged exposure studies had a control and an exposure group. Both studies were conducted thrice separately to confirm reproducibility of SVEE-induced behavior and learning-memory deficits. Each study began with a 1 week acclimatization, followed by a 2 week exposure to vehicle exhaust/normal air, and finally followed by behavioral and cognitive analysis to assess anxiety-, depression-like behavior and learning-memory impairment (Supplementary Fig. S1).

### Simulated vehicle exhaust exposure (SVEE) model

SD rats were housed in standard cages in the climate-controlled rodent housing facility on a 12-h light/dark cycle with *ad libitum* food and water. Experiments were conducted in accordance with the NIH guidelines using protocols approved by the University of Houston Animal Care and Use Committee. The Simulated Vehicle Exhaust Exposure apparatus consisted of two air-tight chambers capable of housing 3-5 rats per chamber (CH Technologies, NJ, USA). Number of rats housed per cage in the rodent housing facility was equal to number of rats housed per chamber of the apparatus during exposure. Each chamber was fitted with a tubing system to allow inflow and outflow of gaseous mixtures (Fig. 1). A regulated valve enabled mixing of toxic gases with air. A flow regulator displayed the flow rate of the gaseous mixture entering the chambers. The simulated mixture of pro-oxidant gases was obtained from Scott Specialty Gases (SCOTT^TM^). The composition of mixture was based upon hazardous constituents found in crude emissions from raw gasoline exhaust (13% CO_2_, 1000 ppm NO_2_ and 0.68% CO in air)^4, 5^ and was further diluted to 1:10 (high dose) or ~1:1000 (low dose) with air. This mixture was devoid of any particulate matter or hydrocarbons. Depending on the study type, EXP rats were subjected to high/low level of simulated exhaust for 30 min/5 h daily for 2 weeks respectively. An important aspect of this model is that the regimen used here is comparable to daily exposure of exhaust levels in areas of high traffic and airports with maximum exhaust emissions^11, 23^. CON groups were exposed to normal air (used as base for the exhaust mixture) for the same duration.

### General Body Parameters

Body weight, food and water intake of rats was monitored throughout the period of exposure to determine the effect of SVEE on general body parameters.

### Behavioral and Cognitive Analysis

All behavioral and learning-memory function tests were performed blind and conducted in the order as indicated in Supplementary Fig. S1.

### Anxiety-Like Behavior Tests

First, open-field test was conducted followed by elevated-plus maze, light dark and marble burying tests as previously published by our lab^8, 24^.

#### Open field test (OFT)

Each rat was placed for 15 min in an OFT apparatus that consisted of an open arena (17.5″ × 17.5″) surrounded by transparent plexiglass walls. Rat was placed in the center of the arena and allowed to move freely in the arena for 15 minutes while its movement was recorded using infrared light sensors as previously published^24^. The time spent in the center of the arena was analyzed and reported as percentage. Reduced percentage of time in the center is an indicator of anxiety-like behavior.

#### Elevated plus maze (EPM) test

Each rat was placed on an EPM apparatus that consisted of four arms (two open and two closed) intersecting in a way that created a plus shape at an elevation^8, 10^. Movement of rat between the arms was recorded for 5 min. Reduced time spent by a rat in the open arm is an indication of anxiety-like behavior.

#### Light-dark (LD) test

Each rat was placed for 5 min in an LD box made of two compartments: a lit compartment and a dark compartment separated by a single partition with an opening to facilitate inter-compartment movement^10^. Time spent in lit area was recorded. Less time spent in the lit compartment suggests increase in anxiety-like behavior.

#### Marble burying (MB) test

Each rat was placed in a cage with marbles on the bedding for 30 min. The rats with higher anxiety levels have tendency to engage in a digging behavior resulting in more number of buried marbles. Therefore, more the number of marbles buried, the higher the anxiety-like behavior^25^.

### Depression-Like Behavior Tests

Forced swim test (FST) was used to assess depression-like behavior in rats. The test was performed as previously published by our lab^24^. Each rat was placed in a cylindrical water tank for 5 min. Time spent immobile is an indicator of depression-like behavior. More time the rat spends immobile, more is the depression-like behavior^24^.

### Learning and Memory-function tests

The radial arm water maze (RAWM) test was performed to assess learning and memory function. Short and long-term memory tests were performed as described in our previous publications^24, 26^. More errors scored in locating the hidden platform are an indication of impaired memory.

### Statistical Analysis

All values were expressed as mean ± SEM. Significance was determined by Student’s t-test (GraphPad Software, Inc. San Diego, CA) since all comparisons were made between 2 independent groups (CON and EXP). For general body measurements where parameters were measured in the same rats at different time intervals; a repeated measures ANOVA test was performed to compare the parameters with time and group as factors. A value of p < 0.05 was considered significant.

### Compliance with ethical standards

All animal studies were conducted in accordance with the NIH guidelines using protocols approved by the University of Houston Animal Care and Use Committee. The manuscript does not contain clinical studies or patient data.

## Electronic supplementary material

Schematic representation of experimental design
