# Supplementary material for: Psychological Impact of Vehicle Exhaust Exposure: Insights from an Animal Model
Source: Sci Rep. 2017 Aug 16;7:8306. doi: 10.1038/s41598-017-08859-1 (PMC5559575; doi:10.1038/s41598-017-08859-1)
Supplement: Supplementary file 1 — Schematic representation of experimental design [file 41598_2017_8859_MOESM1_ESM.pdf]

Psychological Impact of Vehicle Exhaust Exposure: Insights from an Animal Model

Ankita Salvi, Gaurav Patki, Hesong Liu and Samina Salim*

From the Department of Pharmacological and Pharmaceutical Sciences, College of Pharmacy, University of Houston, Houston, TX-77204

* To whom correspondence should be addressed: Samina Salim, Ph.D., Department of Pharmacological and Pharmaceutical Sciences, University of Houston, Texas, USA. Tel: +1 713-743-1776 or 1597; Fax: +1 713-743-1229; Email address: ssalim2@central.uh.edu (Samina Salim)

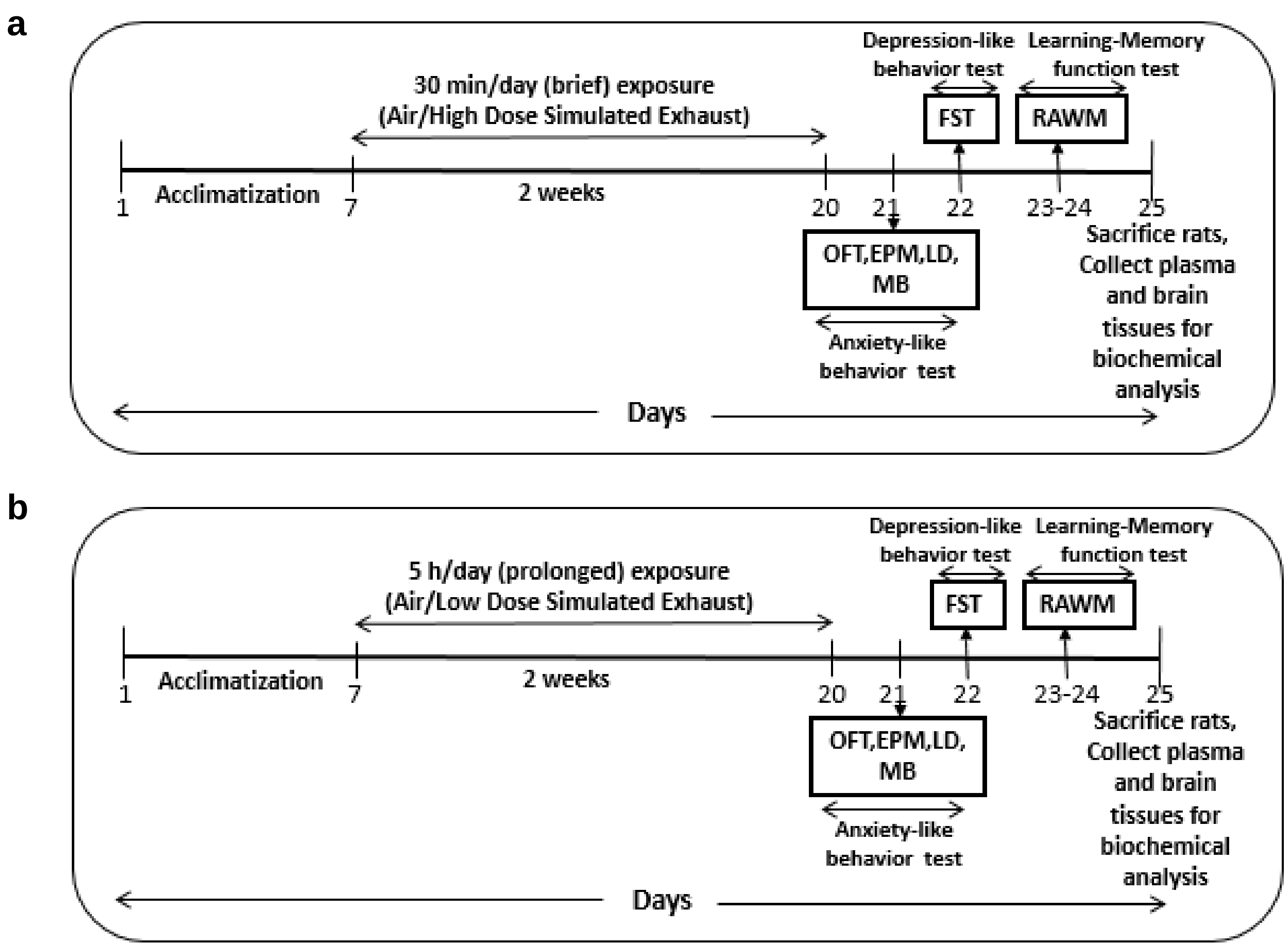

Electronic Supplementary Figure 1:
Schematic representation of the experimental design for high dose brief (a) and low dose prolonged (b) 2 week SVEE
